# Supplementary material for: Integrated analysis of the aqueous humor microbiome and lens capsule transcriptome in high myopia cataract: a pilot study
Source: Front Med (Lausanne). 2026 Jun 16;13:1845205. doi: 10.3389/fmed.2026.1845205 (PMC13314463; doi:10.3389/fmed.2026.1845205)
Supplement: Supplementary file 3 [file Data_Sheet_1.zip › 5.Association/corheatmap/C372089/CorHeatmap.pdf]

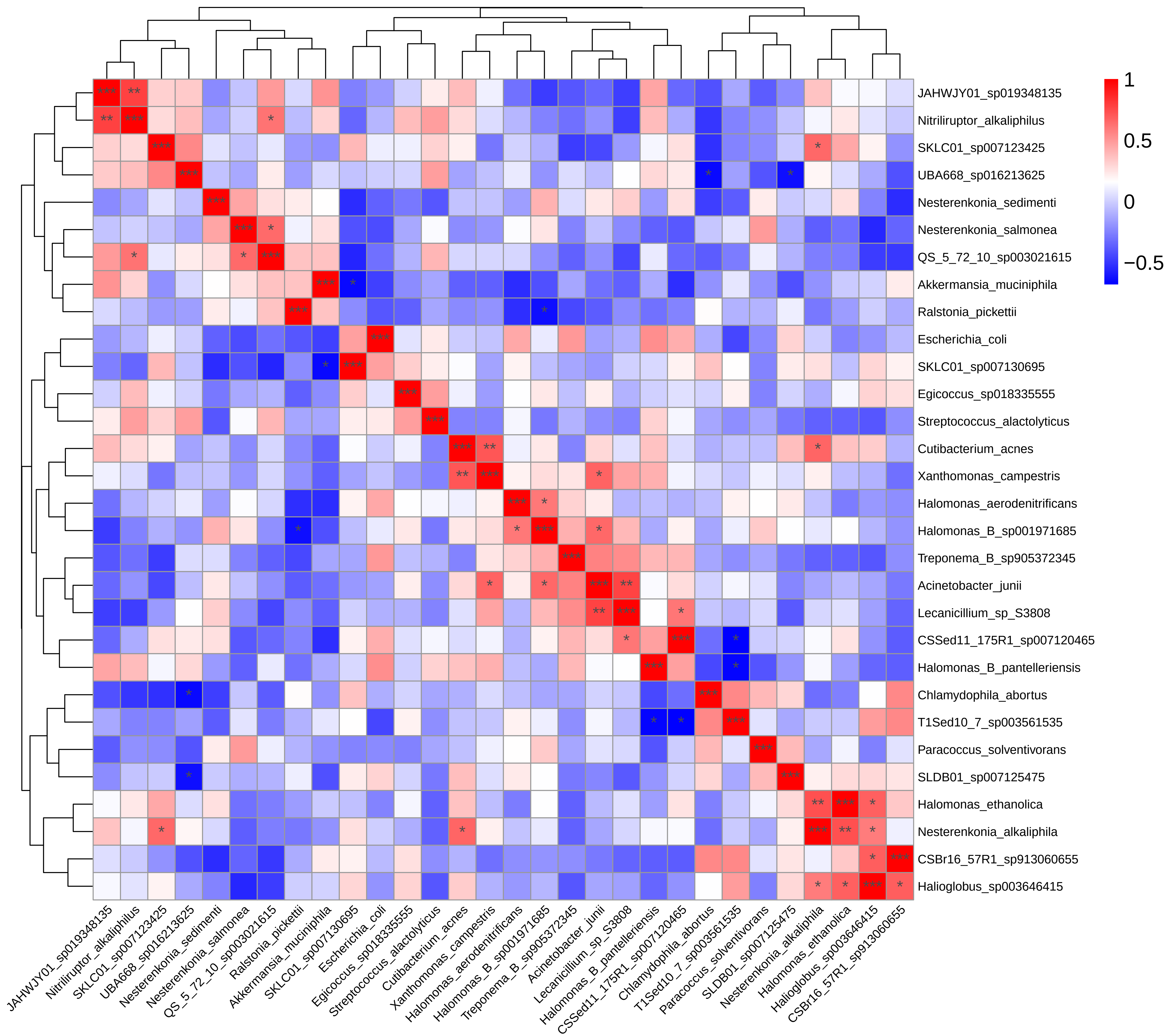
JAHWJY01_sp019348135
Nitriliruptor_alkaliphilus
SKLC01_sp007123425
UBA668_sp016213625
Nesterenkonia_sedimenti
Nesterenkonia_salmonea
QS_5_72_10_sp003021615
Akkermansia_muciniphila
Ralstonia_pickettii
Escherichia_coli
SKLC01_sp007130695
Egicoccus_sp018335555
Streptococcus_alactolyticus
Cutibacterium_acnes
Xanthomonas_campestris
Halomonas_aerodenitrificans
Halomonas_B_sp001971685
Treponema_B_sp905372345
Acinetobacter_junii
Lecanicillium_sp_S3808
CSSed11_175R1_sp007120465
Halomonas_B_pantelleriensis
Chlamydophila_abortus
T1Sed10_7_sp003561535
Paracoccus_solventivorans
SLDB01_sp007125475
Halomonas_ethanolica
Nesterenkonia_alkaliphila
CSBr16_57R1_sp913060655
Halioglobus_sp003646415
1
0.5
0
−0.5
